# LAPTM4B-35, a Cancer-Related Gene, Is Associated with Poor Prognosis in TNM Stages I-III Gastric Cancer Patients

**DOI:** 10.1371/journal.pone.0121559

**Published:** 2015-04-07

**Authors:** Xiaojing Cheng, Zhixue Zheng, Zhaode Bu, Xiaojiang Wu, Lianhai Zhang, Xiaofang Xing, Xiaohong Wang, Ying Hu, Hong Du, Lin Li, Shen Li, Rouli Zhou, Xian-Zi Wen, Jia-Fu Ji

**Affiliations:** 1 Gastrointestinal Carcinoma Translational Research Laboratory, Key Laboratory of Carcinogenesis and Translational Research (Ministry of Education), Peking University Cancer Hospital & Institute, Beijing, China; 2 Department of Gastrointestinal Surgery, Key Laboratory of Carcinogenesis and Translational Research (Ministry of Education), Peking University Cancer Hospital & Institute, Beijing, China; 3 Biological Tissue Bank, Key Laboratory of Carcinogenesis and Translational Research (Ministry of Education), Peking University Cancer Hospital & Institute, Beijing, China; 4 Department of Cell Biology, School of Basic Medical Sciences, Peking University, Beijing, China; Shiga University of Medical science, JAPAN

## Abstract

**Background:**

Lysosome-associated transmembrane protein 4β-35 (LAPTM4B-35), a member of the mammalian 4-tetratransmembrane spanning protein superfamily, has been reported to be overexpressed in several cancers. However the expression of LAPTM4B-35 and its role in the progression of gastric cancer (GC) remains unknown. The aim of this study was to investigate LAPTM4B-35 expression in GC, its potential relevance to clinicopathologic parameters and role of LAPTM4B-35 during gastric carcinogenesis.

**Methods:**

In the present study, paraffin-embedded specimens with GC (n = 240, including 180 paired specimens) and 24 paired fresh frozen tissues were analyzed. qRT-PCR and immunohistochemistry (IHC) were used to analyze the expression of LAPTM4B-35 in GC. The effects of LAPTM4B-35 on GC cell proliferation, migration and invasion were determined by overexpression and knockdown assays.

**Results:**

IHC showed that LAPTM4B-35 was expressed in 68.3% (123/180) of GC tissues, while in 16.1% (29/180) of their paired adjacent noncancerous gastric tissues (*P* = 0.000). *LAPTM4B-35* mRNA levels in GC tissues were also significantly elevated when compared with their paired adjacent noncancerous tissues (*P* = 0.017). Overexpression of LAPTM4B-35 was significantly associated with degree of differentiation, depth of invasion, lymphovascular invasion and lymph node metastasis (*P*<0.05). Kaplan-Meier survival curves revealed that patients with LAPTM4B-35 expression had a significant decrease in overall survival (OS) in stages I-III GC patients (*P* = 0.006). Multivariate analysis showed high expression of LAPTM4B-35 was an independent prognostic factor for OS in stage I-III GC patients (*P* = 0.025).

**Conclusion:**

These findings indicate that LAPTM4B-35 overexpression may be related to GC progression and poor prognosis, and thus may serve as a new prediction marker of prognosis in GC patients.

## Introduction

Gastric cancer is the third most common cancer in China and the leading cause of cancer-related death in China [1]. The majority of GC patients are already in advanced stage at the time of diagnosis (stage III and IV), rendering the prognosis to be dismal, despite improving surgical and adjuvant treatment approach [2–5]. Regardless of chemotherapy and intended curative surgery, nearly 60% of patients with GC develop recurrence and eventually die of metastatic disease [6]. GC progression is a multifactorial and multistep process in which inherited and environmental factors play a vital roles [7]. Previous observations indicate that classic biomarkers and staging systems, based on clinical and pathologic findings, might have their limitations on clinical applications and impel to develop new molecular biomarkers which can predict patient outcome and treatment [8, 9].

Lysosome-associated protein transmembrane 4 beta (LAPTM4B) has been originally cloned in hepatocellular carcinomas (HCCs) by Shao et al, which located at chromosome 8q22, a region frequently amplified in breast cancer and HCC [10, 11]. It encodes two proteins with different molecular weight, 24-kDa and 35-kDa proteins (LAPTM4B-24 and -35) with four putative transmembrane regions [12]. The localization of LAPTM4B-35 was found not only in lysosome, but also in plasma membrane and internal organelles such as Golgi apparatus and endosomes [13]. It has been reported that LAPTM4B-35 was widely expressed in various types of carcinoma. Previous reports indicated that overexpression of LAPTM4B-35 is associated with unfavorable biological behaviors and poor prognosis in many cancers, such as breast cancer [14], HCC [15–17], gallbladder carcinoma [18, 19], colorectal cancer [20], epithelial ovarian carcinoma [21] and endometrial carcinoma [22]. It also has been reported that allelic variation of LAPTM4B was associated with genetic susceptibility of GC [23]. Moreover, overexpression of LAPTM4B-35 by transfection of LAPTM4B cDNA promotes cell proliferation, migration, invasion in HCC xenografts in nude mice and induces multidrug resistance [24]. Knockdown of LAPTM4B by RNA interference conversely reversed all of these malignant phenotypic features in HCC [24, 25].

However, LAPTM4B-35 expression, its relevance to the clinicopathologic characteristics, and biological role of LAPTM4B-35 in GC remain unclear. In the present study, we aimed to identify LAPMT4B-35 expression in GC and its potential relationship with clinicopathological features, and its prognostic significance. In addition, in vitro functional assays were performed to characterize the biological effects of LAPTMEB-35 in gastric tumorigenicity.

## Materials and Methods

### Ethics

All patients had signed informed consent for obtaining tissue samples, and the study protocol was approved by the Clinical Research Ethics Committee of Peking University Cancer Hospital.

### Human gastric tissue specimens

A total of 240 (167 males and 73 females, aged 22–87 years, and the median age was 60 years) formalin fixed paraffin embedded GC tissues, including 180 paired cancerous and matched adjacent noncancerous gastric mucosa tissues, were collected from GC patients undergoing gastrectomy at Peking University Cancer Hospital from January 2003 to December 2011. The clinical and histological information for each case was also collected according to approved institutional guidelines. The 1997 UICC-TNM criteria were used for classification of gastric cancers. The median follow-up duration since the time of diagnosis was 26.2 months (range, 2.4–119.0 months). In total, 112 (46.7%) patients died in the follow-up period.

### Immunohistochemistry

Paraffin sections (4 μm) were dewaxed and rehydrated in xylem and ethanol. Immunohistochemistry was performed as previously described [16] using anti-LAPTM4B-35 antibody (1:200) (gifted by Pro. RL Zhou). As negative controls, the sections were processed as the same protocol except that they were incubated overnight at 4°C in blocking solution without the primary antibody.

The expression of LAPTM4B-35 was assessed by two experienced pathologists (Y Sun and B Dong), who worked independently and were blinded to the patients’ clinical outcomes. Discrepancies between the observers were found in less than 10% of the examined slides, and a consensus was reached after further review. Staining assessment was just used the semiquantitative methods to classify LAPTM4B-35 expression as negative and positive stained immunoreactive cells. The ratio of positivity was scored as ‘‘0” (<10% positive tumor cells), ‘‘1” (10–50% positive tumor cells), and ‘‘2” (>50% positive tumor cells). The staining intensity was scored as ‘‘0” (no staining or weakly stained), ‘‘1” (moderate staining), or ‘‘2” (strong staining). The sum of the staining intensity score and the percentage score was used to define the LAPMT4B expression levels: 0–2, negative expression and 3–4, positive expression (14). The positive expression only evaluated by the sections with at least 10% positive area of tumors.

### Cell culture, LAPTM4B-35 expression plasmid and hCas9/gRNA transfection

Gastric cancer cell lines (SGC-7901, BGC-823, MGC-803, MKN-28 and AGS) were cultured in DMEM (Wisent Inc., St-Bruno, QC, Canada) supplemented with 10% fetal bovine serum (FBS) (HyClone, Logan, UT, USA) and 100 U/ml penicillin and 100 μg/ml streptomycin at 37°C in an atmosphere of 5% CO_2_. The *pcDNA3*.*0-AF* containing the whole ORF of LAPTM4B producing LAPTM4B-35 protein was gifted by Prof. RL Zhou [11]. The transfection was performed with Lipofectamine 2000 (Invitrogen, Carlsbad, CA) according to the manufacturer’s protocol. Positive cell clones were obtained by antibiotic selection with G418 (Gibco, Grand Island, NY) at a concentration of 800 ng/ml.

hCas9 and gRNA vector were gotten from China Zebrafish Resource Center (CZRC). The full length of Cas9 cDNA was cloned into the pXT7 vector and linearized, and capped mRNA was synthesized using mMESSAGE mMACHINE mRNA transcription synthesis kits (Life Technologies, Carlsbad, California, USA). The gRNA primer sequences used were: forward primer, 5’-TACGACTCACTATAGGGGGATGGTGCCGGTGCGGACAGTTT TAGAGCTAGAAATAGC-3’, and reverse primer: 5’-AGCACCGACTCGGTGCCACT-3’.The protocol was followed by the previous published paper [26]. hCas9 mRNA (3 ng/μl) and gRNA (0.2 ng/μl) were cotransfected into SGC-7901 cell performed with Lipofectamine 2000 (Invitrogen, Carlsbad, CA).

### RNA isolation, quantitative real-time reverse transcription PCR (qRT-PCR)

Total RNA was extracted using TRIzol reagent (Invitrogen, Carlsbad, CA, USA) and reverse transcribed into cDNA using M-MLV reverse transcriptase (Promega, Madison, WI, USA) according to the manufacturer’s instructions. Real-time PCR was performed using the ABI 7500 Fast real-time PCR system (Life Technologies, Carlsbad, California, USA). β-actin was used as an internal control. The primer sequences used are listed below: LAPMT4B-35: forward primer: 5’-GGAAGCAGGACAGCCAACTT-3’, reverse primer: 5’-TTATTCTCGATCTCACAACCAAAC-3’; β-actin: forward primer: 5’- CCTGTGGCATCCACGAAACT-3’ reverse primer: 5’- GAAGCATTTGCGGTGGACGAT-3’.

### Cell proliferation assay

Cell proliferation was measured by Cell Counting Kit-8 (CCK-8) (Dojindo, Japan) according to the manufacture’s protocol. A volume of 100μl of cell suspension (3000 cells per well) was incubated in a 96-well plate (Costar, Corning, NY) for 24, 48, 72, 96 hrs. 10μl of the CCK-8 solution was added to each well and incubated for 3 hrs at 37 °C. Absorbance values of all wells were then determined at 450nm with a reference wavelength of 630nm in Microplate Reader (Bio-Rad, USA). The experiment was done in triplicate and repeated twice.

### Wound-healing assay

Cell migration was measured by wound-healing assay by the IncuCyte HD system (IncuCyte ZOOM, Essen BioScience, USA). Cells were seeded in 96-well plates at the density of 6×10^4^ cells per well. Wound was made through confluent monolayer cells with a pin block and cells were washed with 1×PBS, cultured in DMEM medium with 10% fetal bovine serum. Photographs of cells were imaged using the IncuCyte HD system at 4-hour intervals from 2 separate regions per well using a 10x objective. Values from 2 regions of each well were pooled and averaged across all 3 replicates.

### Transwell invasion assays

The Transwell (8μm pore size; Cell Biolabs, USA) assay was used to analyze cell invasion according to the manufacturer’s instructions. 8×10^4^ cells were placed in the upper chambers in serum-free media, and the lower chambers were filled with DMEM and 10% FBS. Following incubation for 72 hrs at 37°C, non-invasive cells on the top surface of the membranes were removed with a cotton swab. The membranes were fixed with methanol for 10 min and stained with 0.5% crystal violet for 10 min. The cells on the underside of the filter were from five randomly selected microscopic views were counted.

### Western blot analysis

The protein expression levels of GC cell lines were measured by Western Blot analysis. Cells were lysed in pre-chilled RIPA lysis buffer (Pierce Biotechnology, Rockford, IL) containing protease inhibitor cocktail (Roche, Basel, Switzerland) for 30 min after centrifugation at 15,000g for 20 min, the supernatant was obtained. 50μg of protein extracts were separated by 10% SDS polyacrylamide gel electrophoresis, and transferred onto the 0.45μm polyvinylidene difluoride (PVDF) membrane (Whatman, Germany). The membrane was blocked for 1 hr at room temperature with blocking buffer (pH 7.6) containing 5% nonfat dry milk, then incubated with anti-LAPTM4B-35 antibody (1:800; gifted by Prof. RL Zhou) at 4°C overnight. Mouse anti-human β-actin antibody (Santa Cruz Biotechnology, Santa Cruz, CA) was applied as an internal control. Immunoreactive bands were visualized using a chemiluminescence detection system (Pierce Biotechnology, Rockford, IL).

### Statistical analysis

Chi-square test was used to compare the difference in LAPTM4B-35 protein expression between GC tissues and adjacent noncancerous tissues. Unconditional logistic regression analysis models were used to analyze relationships between LAPTM4B-35 expression and clinicopathological parameters adjusted by gender status. The difference of *LAPMT4B-35* mRNA expression between GC and adjacent noncancerous tissues was analyzed by the Wilcoxon matched pair test.

Overall survival (OS) curve was calculated with the Kaplan-Meier method and analyzed with the log-rank test. Relative risks (RRs) of death associated with LAPTM4B-35 expression and other predictor variables were estimated from the univariate Cox proportional hazards model firstly. Multivariate Cox models also were constructed to estimate the RR for LAPMT4B-35 expression. All statistical analyses were carried out using the SPSS software statistical package (version 20.0; SPSS Inc., Chicago, IL, USA). A two-sided *P* value less than 0.05 was considered as statistical significance.

## Results

### Expression analysis of LAPTM4B-35 in GC cell lines and GCs

**Fig 1:**
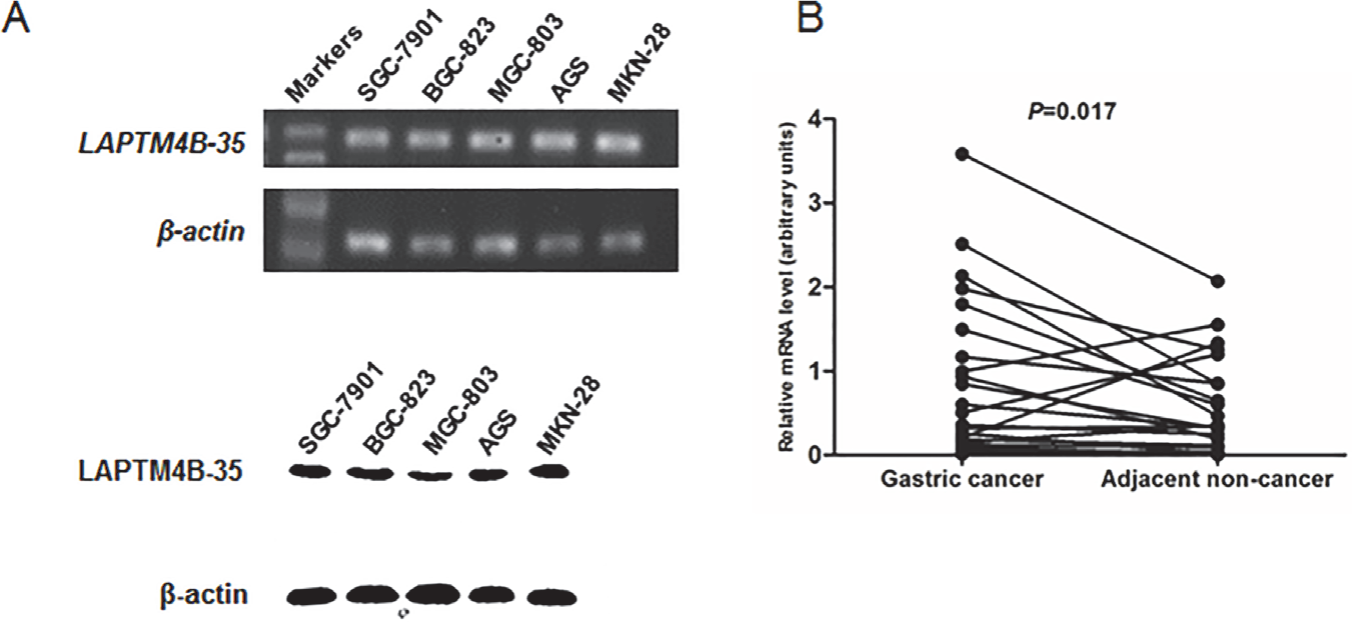
Expression analysis of LAPTM4B-35 in GC cell lines and GCs. (A) mRNA (upper panel) and protein (lower panel) expression of LAPTM4B-35 in five gastric cancer cell lines. (B) relative mRNA expression of *LAPTM4B-35* in gastric cancer and their adjacent noncancerous tissues.

We firstly examined the expression of *LAPTM4B-35* by RT-PCR in 5 GC cell lines (MGC-803, BGC-823, MKN-28, SGC-7901 and AGS), and then detected its protein expression by Western blot. The results showed that LAPTM4B-35 was expressed in all the cell lines in both mRNA and protein levels (Fig 1A, upper and lower panel), then we chose BGC-823 and SGC-7901 cells for our following cell function tests.

*LAPTM4B-35* expression was also detected in 24 pairs of resected GC specimens by qRT-PCR. As we can see in the Fig 1B, the *LAPTM4B-35* expression in GC tissues was significantly elevated when compared with paired adjacent noncancerous tissues (*P* = 0.017) (Fig 1B).

### LAPTM4B-35 protein expression was frequently observed in human GC

**Fig 2:**
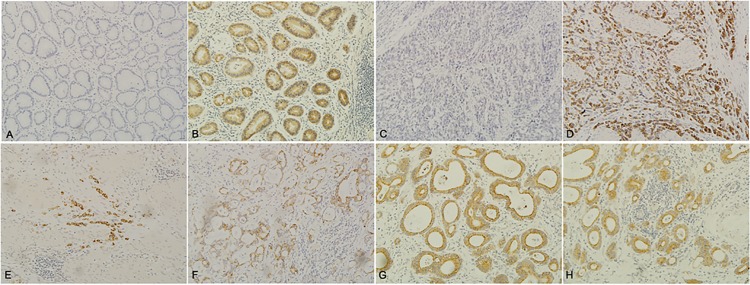
Immunohistochemical staining with anti-LAPTM4B-35 protein in normal, noncancerous and carcinomas of the stomach. (A) LAPTM4B-35 was not expressed in normal stomach mucosa. (B) LAPTM4B-35 was expressed in the dysplasia lesion. (C) LAPTM4B-35 negative staining in GC tissue. (E-H) LAPTM4B-35 positive staining in GC tissues. Original magnification: 100×.

We investigated the expression of LAPTM4B-35 in GC and adjacent noncancerous tissues by means of immunohistochemical analysis. LAPTM4B-35 did not express in normal gastric mucosa (Fig 2A), but expressed in the intestinal metaplasia、dysplasia lesion (Data was not shown) and tumor cells, and mainly localized within the cytoplasm or on the cell membrane (Fig 2B and 2D-2H). LAPTM4B-35 frequently observed in GC tissues compared with matched adjacent noncancerous mucosa (68.3% vs. 16.1%, *P* = 0.000) (Table 1).

**Table 1:** Expression of LAPTM4B-35 in Gastric Specimens by immunnohistochemistry.

Variable	LAPTM4B-35 expression		***P*** * value
	Negative (%)	Positive (%)	
Gastric cancer tissues	57 (31.7%)	123 (68.3%)	0.000
Adjacent noncancerous tissues	151 (83.9%)	29 (16.1%)	

* Chi-square test

### Relationship between LAPTM4B-35 expression and clinicopathological characteristics

We analyzed the relationship between LAPTM4B-35 expression and clinicopathological characteristics of GC patients. As the gender, the objects in our study has their deviation (167 vs. 73, Table 2), we calculated their relationship by logistic regression analysis and adjusted for gender status. LAPTM4B-35 was more frequently observed poorly differentiated GCs compared with moderate-well differentiated ones (65.4% vs. 57.9%, *P* = 0.017). Furthermore, LAPTM4B-35 expression was associated with lymphovascular invasion (*P* = 0.000), depth of invasion (*P* = 0.016) and lymph node metastasis (*P* = 0.029) (Table 2).

**Table 2:** Relationship between LAPTM4B-35 Expression and Clinicopathological Features of Patients with Gastric Cancer.

Clinicopathological Features	LAPTM4B-35 expression		***P*** ^a^ value	***P*** ^b^ value
	Negative (%)	Positive (%)		
Gender				
Male	37 (22.2%)	130 (77.8%)	0.001	0.000
Female	32 (43.8)	41 (56.2%)		
Age, year				
≤60	40 (32.8%)	82 (67.2%)	0.160	0.476
>60	29 (24.6%)	89 (75.4%)		
Tumor location				
Upper 1/3	6 (11.5%)	46 (88.5%)	0.000	0.142
Middle 1/3	12 (21.8%)	43 (78.2%)		
Low 1/3	46 (36.2%)	81 (63.8%)		
Multiple site	5 (83.3%)	1 (16.7%)		
Tumor size				
≤4cm	38 (30.2%)	88 (69.8%)	0.612	0.612
>4cm	31 (27.2%)	83 (72.8%)		
Lauren				
Intestinal	41 (25.9%)	117 (74.1%)	0.183	0.184
Diffuse/mixed	28 (34.1%)	54 (65.9%)		
Differentiation				
Well-Moderate	24 (42.1%)	33 (57.9%)	0.011	0.017
Poor	45 (24.6%)	138 (75.4%)		
Histologic type				
Adenocarcinoma	51 (27.1%)	137(72.9%)	0.291	0.002
Others^δ^	18(34.6%)	34(65.4%)		
Lymphovascular invasion				
Absent	37 (30.8%)	83 (69.2%)	0.476	0.000
Present	32 (26.7%)	88 (73.3%)		
Depth of invasion				
T1	7 (50.0%)	7 (50.0%)		0.016^ϕ^
T2	10 (38.5%)	16 (61.5%)		
T3	15 (29.4%)	36 (70.6%)		
T4	37 (24.8%)	112 (75.2%)		
Lymph node metastasis				
No	22 (40.7%)	32 (59.3%)	0.027	0.029
Yes	47 (25.3%)	139 (74.7%)		
Distant metastasis				
M0	56 (27.3%)	149 (72.7%)	0.235	0.238
M1	13 (37.1%)	22 (62.9%)		
TNM stage				
I	14 (56.0%)	11 (44.0%)	0.005	0.530^ϕ^
II	14 (23.3%)	46 (76.7%)		
III	28 (24.2%)	92 (75.8%)		
IV	13 (37.1%)	22 (62.9%)		

^a^ Chi-square test;

^b^Logistic regression: Data were calculated by logistic regression analysis and adjusted by gender status;

^δ^ signet-ring cell carcinoma and mucinous adenocarcinoma;

^ϕ^ linear correlation coefficient

### LAPTM4B-35 expression and prognosis of GC patients

**Fig 3:**
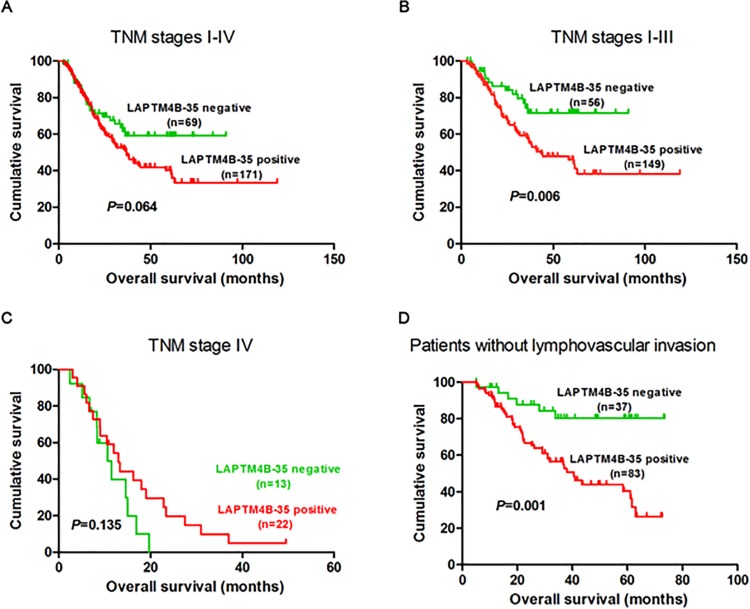
Kaplan-Meier curves for overall survival of the GC patients. **A,** Kaplan-Meier survival curves for OS in GC patients with LAPTM4B-35 positive and negative expression. (B, C, D) Kaplan-Meier curves for OS in subgroup of GC patients stratified by TNM stages I-III, IV and patients without lymphovascular invasion, respectively.

Fig 3 depicts the analysis of LAPTM4B-35 expression and clinical outcomes. Kaplan-Meier survival curves showed there was a tendency of shorter overall survival (OS) in GC patients with LAPTM4B-35 positive expression compared to the negative ones (*P* = 0.064, log-rank = 3.425) (Fig 3A). After patients were stratified by TNM I-III or patients without lypmphovascular invasion, patients with LAPTM4B-35 positive expression showed significantly shorter OS than those negative ones (*P* = 0.006 and *P* = 0.001, respectively) (Fig 3B and 3D).

The results of univariate and multivariate Cox’s models for OS of GC patients in TNM Stage I-III exhibited that depth of invasion (RR = 3.103, 95% CI: 1.427–6.748; *P* = 0.004), lymph node metastasis (RR = 3.015, 95%CI: 1.552–5.858; *P* = 0.001) and LAPTM4B-35 expression level (RR = 2.249, 95%CI: 1.242–4.072; *P* = 0.007) significantly affected the survival of GC, respectively (Table 3); furthermore, LAPTM4B-35 positive expression was an independent prognostic factor (RR = 1.897, 95%CI: 1.041–3.457; *P* = 0.025). Lymph node metastasis (RR = 2.665, 95%CI: 1.362–5.213; *P* = 0.001) also independently predicted OS (Table 3).

**Table 3:** Results of Univariate and Multivariate Cox’s models for OS of GC patients in TNM Stage I-III.

Variables	Univariate analysis			Multivariate analysis		
	RR	95% CI	***P*** ^a^ value	RR	95% CI	***P*** ^b^ value
Gender						
Male vs. Female	0.600	0.355–1.014	0.056			
Age, year						
≤60 vs. >60	1.386	0.891–2.155	0.148			
Tumor location						
Upper 1/3	1.000		0.061			
Middle 1/3	0.479	0.144–1.593	0.230			
Low 1/3	0.378	0.111–1.279	0.118			
Multiple site	0.279	0.086–0.908	0.034			
Tumor size						
≤4cm vs. >4cm	1.488	0.959–2.308	0.076			
Lauren						
Intestinal vs. Diffuse/mixed	1.407	0.899–2.203	0.135			
Degree of differentiation						
Well-Moderate vs. Poor	1.582	0.903–2.773	0.109			
Histology type						
Adenocarcinoma vs. Others	0.809	0.461–1.417	0.458			
Lymphovascular invasion						
Absent vs. Present	1.222	0.791–1.890	0.367			
Depth of invasion						
T_1_+T_2_ vs. T_3_+T_4_	3.103	1.427–6.748	0.004			
Lymph node metastasis						
No vs. Yes	3.015	1.552–5.858	0.001	2.665	1.362–5.213	0.001
LAPTM4B-35						
Negative vs. Positive	2.249	1.242–4.072	0.007	1.897	1.041–3.457	0.025

RR, relative risk; CI, confidence interval.

^a^ Log-rank test;

^b^Cox regression test.

### LAPTM4B-35 promoted tumor cell proliferation

**Fig 4:**
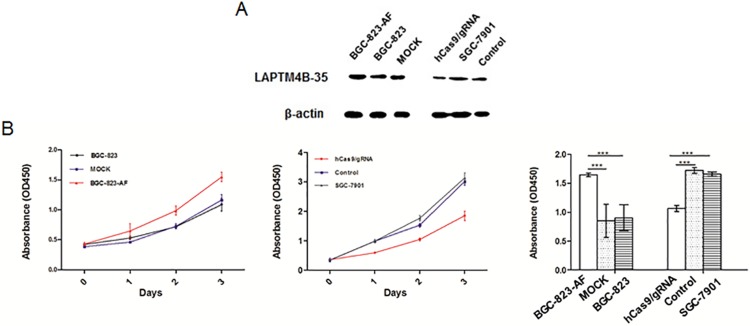
Effect of LAPTM4B-35 on cell proliferation. **(A)** Expression of LAPTM4B-35 in BGC-823 and SGC-7901 after transfection with LAPTM4B expression vector and hCas9/gRNA were identified by Western-blot. BGC-823-AF shows BGC-823-overexpressing clone. (B) Growth curves determined by CCK-8 assay. Left panel: overexpression of LAPTM4B-35 promotes rapid increase in BGC-823 cell proliferation compared with wild-type and MOCK. Middle panel: knockdown of LAPTM4B-35 expression inhibits cell proliferation as compared with control and SGC-7901. Right panel: different cell proliferation state after incubation 3 days for BGC-823 and 2 days for SGC-7901. *P<0.05, BGC-823-AF vs MOCK, knockdown vs SGC-7901. For all data the mean and standard deviation represent the average of three independent experiments.

In order to investigate the effect of LAPMT4B-35 in the function of cell biology, two cell lines were established. BGC-823 and SGC-7901 cells presenting relatively lower or higher expression of LAPTM4B-35 were separately selected for overexpression and knockdown assay (Fig 1A). In the first, cell line BGC-823-AF overexpressing LAPTM4B-35 stably was established, and MOCK was used as the control for this cell line. Second, LAPTM4B-35 was stably knocked down towarding to SGC-7901(hCas9/gRNA) by the type II clustered regularly interspaced short palindromic repeats (CRISPR) system, and the results were identified by Western blot (Fig 4A).

Cell proliferation assay was measured by CCK-8 cell counting kit. The absorbance value of the BGC-823-AF cells at 72 hrs after spreading was significantly higher than those parent cells and MOCK cells, and the result was just opposite by knocking down of LAPTM4B-35 in SGC-7901 cells (Fig 4B).

### LAPTM4B-35 enhanced tumor cell migration and invasion ability

**Fig 5:**
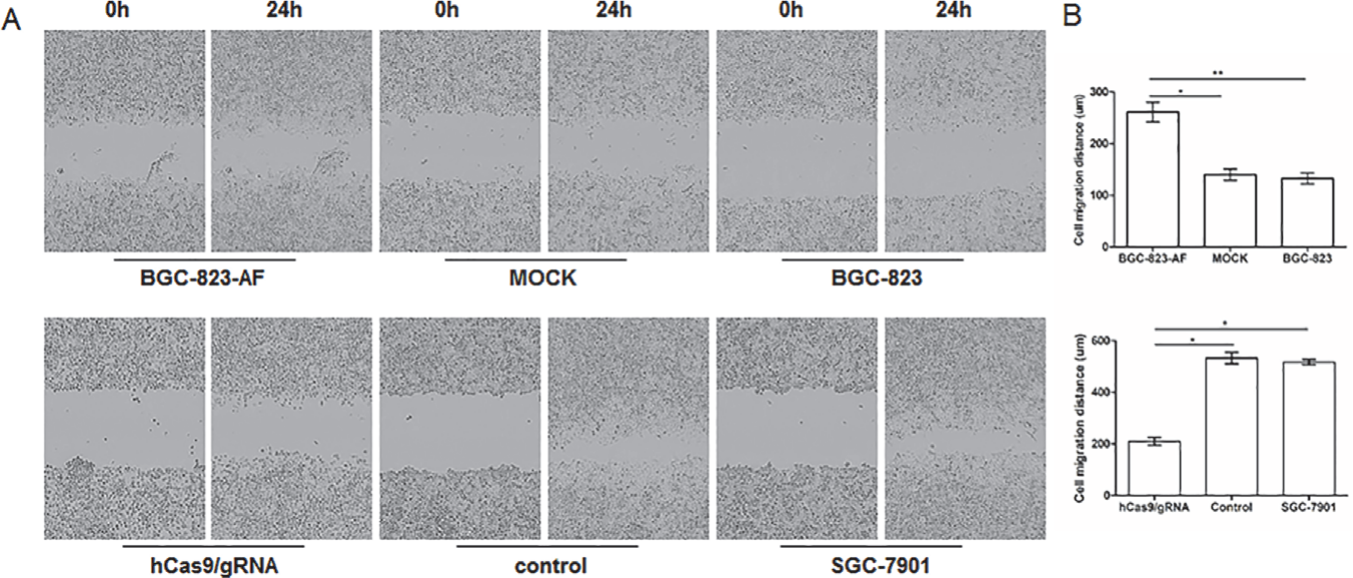
LAPTM4B-35 promotes gastric cancer cells migration. (A) left upper panel: wound-healing assay of BGC-823-AF, MOCK and BGC-823; left lower panel: wound-healing assay of hCas9/gRNA, Control and SGC-7901. Photos were captured by an inverted phase-contrast microscope at 24h after wounding. Magnification = 100×. (B) Quantification of wound-healing rates. For all data the mean and standard deviation represent the average of three independent experiments.

Following transfection with pcDNA3.0-AF and hCas9/gRNA, we performed wound-healing assay to analyze the migration ability of LAPTM4B-35 over-expression/knockdown gastric cancer cells (Fig 5A). Results showed that LAPTM4B-35 promoted BGC-823 (BGC-823-AF) cell migration when compared with BGC-823 and MOCK (*P*<0.05), and down-regulation of LAPTM4B-35 expression could reverse this phenomenon in SGC-7901 cells (*P*<0.05, Fig 5B).

**Fig 6:**
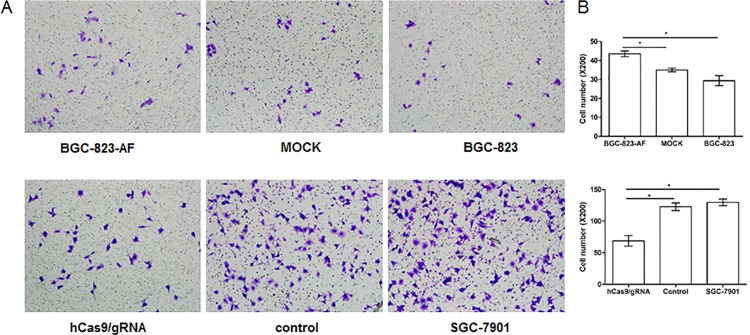
LAPTM4B-35 promotes gastric cancer cells invasion. (A), left upper panel: transwell assay of BGC-823-AF, MOCK and BGC-823 cells; left lower panel: transwell assay of hCas9/gRNA, Control and SGC-7901. Invasive cells were counted in randomly 5 selected microscopic fields. Magnification = 100×. (B) Quantification of migrating and invasive cells. For all data the mean and standard deviation represent the average of three independent experiments.

Accordingly, the effect of LAPTM4B-35 on the invasiveness of tumor cells was studied using the transwell assay (Fig 6A). We found that the invaded cell number was significantly higher in the LAPTM4B-35-transfected BGC-823 cells and lower in the knockdown SGC-7901 cells, than in their control cells, respectively (*P*<0.05, Fig 6B). These results suggest that LAPTM4B-35 promotes the migration and invasion of gastric cancer cells.

## Discussion

GC is a highly heterogeneous disease where even similar clinical and pathological features lead to distinct outcomes [27, 28], indicating that TNM staging system for GC has their limitation and impel to search for new molecular biomarkers which can predict patient’s outcome and treatment. The oncogene LAPTM4B-35 is located on chromosome 8q22, and gain of DNA copy number on chromosome 8q22 is a frequent finding in GC according to our previous study (unpublished data). In breast cancer, overexpression of LAPTM4B-35 and 8q22 amplification were contributed to *de nove* chemoresistance to anthracyclines and are permissive for metastatic recurrence [29].

In this study, we investigated *LAPTM4B-35* expression in 24 gastric cancer and their paired noncancerous gastric surgical specimens by qRT-PCR and found that *LAPTM4B-35* mRNA expression level in gastric cancer was significantly elevated compared with that in the paired noncancerous tissue group. Moreover, immunohistochemical analysis in 180 pairs of gastric cancer showed LAPTM4B-35 was overexpressed in GC tissues (68.3%) compared with their paired noncancerous mucosa (16.1%). LAPTM4B-35 promotes tumor growth and tolerance to metabolic and genetic stress through the induction of autophagy [30, 31]. In our present study, the function of LAPTM4B in gastric cancer was investigated by in vitro assays. Overexpression of LAPTM4B-35 in BGC-823 cells also increased cell viability in serum free culture conditions as well. If this phenomenon is caused by autophage is needed to be investigated.

Furthermore, we also analyzed the correlation of LAPTM4B-35 expression with clinicopathological parameters in gastric cancer. High LAPTM4B-35 expression was significantly correlated with degree of differentiation, lymphovascular invasion, depth of invasion and lymph node metastasis, suggesting that LAPTM4B-35 may be extensively activated in gastric cancer and it may play a vital role in gastric carcinogenesis and tumor progression. As the tumor lymphovascular invasion and lymphonode metastasis are highly important clinical prognostic indicators for tumor recurrence, we made a survival analysis. Kaplan-Meier curves stratified by LAPTM4B-35 expression and tumor stage or lymphovascular invasion revealed that LAPTM4B-35 positive patients have significantly poorer OS compared with LAPTM4B-35 negative ones in TNM stages I-III or without lymphovascular invasion subgroup. Multivariate Cox regression analysis in this subgroup demonstrated that among all analyzed factors, LAPTM4B-35 expression is an independent prognostic factor in gastric cancer patients in TNM stages I-III but in stage IV. It is reasonable because patient in stage IV is unable to receive radical operation and may be treated with many other different palliative treatments, all of which would impact on the prognosis. These results indicate that the propensity for LAPTM4B-35 may specifically predicts the most aggressive and fatal types of gastric cancer in cases with early and without distal metastasis or without lymphovascular invasion.

In our present study, we found that LAPTM4B-35 positive expression generally correlated with worse prognosis in GC patients stratified by tumor stage or lymphovascular invasion. For the function of LAPTM4B-35 in GC, we further performed in vitro assay. Overexpression of LAPTM4B-35 in BGS-823 cells revealed a significant increase in cell proliferation, migration and invasion, and down-regulation of LAPMT4B-35 leaded to the opposite results. Zhou et al. has showed that LAPTM4B-35 overexpression promotes cell survival, deregulated proliferation and migration in HCC cells [12]. Recent studies have reported that LAPTM4B-35 was associated significantly with a worse clinical outcome in many human malignancies, such as HCC [16], metastatic ovarian tumor [32], gallbladder [19, 33], extrahepatic cholangiocarcinoma [34] and endometrial cancer [22]. These accumulated evidences support our current findings, indicating that LAPTM4B-35 overexpression may play an important role in the malignant transformation and progression of GC.

The molecular mechanisms of LAPTM4B-35 in tumor progression properties are still unclear. Some previous studies indicated that LAPTM4B-35 is required for lysosome homeostasis, acidification and function, and that LAPTM4B-35 renders tumor cells resistant to lysosome-mediated cell death triggered by environmental and genotoxic stresses [30, 31]. Overexpression of LAPTM4B-35 could activate some proto-oncogenes, such as c-myc, c-jun and c-fos [35], and promote proliferation, migration, and invasion in some human cancer lines which would enhance the growth and metastasis of HCC xenografts in nude mice [36]. Related investigation implied that LAPTM4B can improve cell proliferation or survival through the involvement in the signal transduction pathway, such as the phosphatidylinositol 3 kinase-protein kinase B pathway. And LAPTM4B-35 may interact with some cancer-related proteins, like protein phosphatase 2A and protein kinase C [10]. Li et al found that LAPTM4B-35 motivates multidrug resistance of cancer cells by promoting drug efflux and anti-apoptosis by activating PI3K/AKT signaling [24], indicating that LAPTM4B-35 may be a new target of therapy. The functional role and mechanism of LAPTM4B in GC need further investigation.

In conclusion, the current study indicates that LPTM4B-35 overexpression plays important roles in promoting cell proliferation, migration and invasion in gastric cancers. LAPTM4B-35 positive expression in gastric cancer tissues correlated with the poor outcome and proved to be an independent prognostic factor in subgroup of GC patient in TNM stages I-III or without lymphovascular invasion. Thus, LAPTM4B-35 may constitute a useful biomarker to predict prognosis in certain subgroup of GC. In addition, as the roles of LAPTM4B-35 in limiting lysosome-mediated cell death and promoting autophagy have significant survival effects in cancer cells, and LAPTM4B-35 was over-expressed in a great proportion of gastric cancer, it might be a helpful target for treating the subtype group of gastric cancer.

## Supporting Information

S1 Dataset Individual results of relative mRNA of LAPTM4B-35 expression, cell proliferation, migration and invasion, survival of pathients and clinicopathological features. (Excel)
(XLS)
